# Monomer Equilibrium and Transport in Emulsion and Miniemulsion Polymerization

**DOI:** 10.1021/acs.biomac.4c00412

**Published:** 2024-05-22

**Authors:** F. Joseph Schork

**Affiliations:** School of Chemical and Biomolecular Engineering Georgia Institute of Technology, Atlanta, Georgia 30332, United States

## Abstract

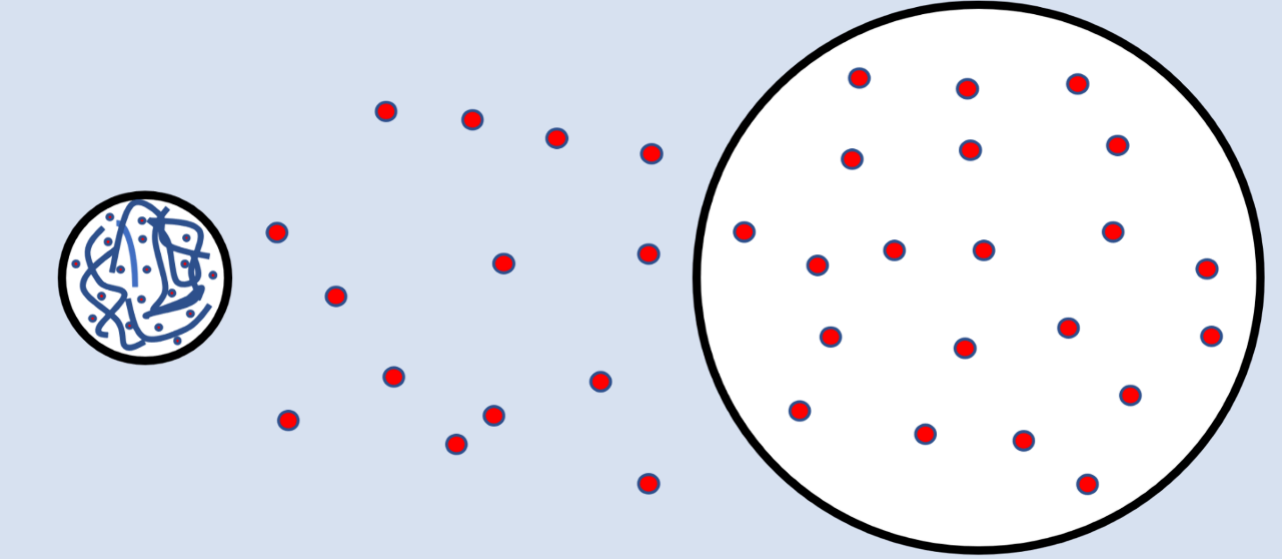

The dual concepts of monomer equilibrium and monomer transport in emulsion and miniemulsion polymerization are discussed in depth. The concepts must be considered together since, first, dispersed-phase polymerizations (in this case emulsion and miniemulsion polymerizations) are by their nature multiphase systems that function only due to interphase mass transfer; and second, because phase equilibrium determines the driving force for monomer transport in these (and all heterophase reaction) systems. Concepts of polymer particle swelling are reviewed, and the question first addressed by Paul Flory in the 1950s: *Are emulsion polymerizations transport or reaction limited?* is revisited in detail.

## 1 Introduction

### 1.1 Dispersed-Phase Polymerization

Free radical polymerization of vinylic monomers can take place in a number of what are called dispersed-phase polymerization systems. These include emulsion polymerization, inverse emulsion polymerization, dispersion polymerization, precipitation polymerization, suspension polymerization, microsuspension polymerization, miniemulsion polymerization, inverse miniemulsion polymerization, and microemulsion polymerization. The most commercially important of these is emulsion polymerization. However, in discussing monomer equilibrium and transport in emulsion polymerization, it will be instructive to look at miniemulsion polymerization as well. What follows is intended to be an in-depth discussion of these phenomena, but not an exhaustive review of previous work.

#### 1.1.1 Emulsion Polymerization

Conventional emulsion polymerization^1^ consists of water-insoluble vinylic monomer (e.g., styrene [STY]) emulsified into an aqueous continuous phase with an oil-in-water surfactant. The process is used to produce coatings and elastomers. A conventional batch emulsion polymerization reaction can be divided into three intervals. Particle nucleation occurs during Interval I and is usually completed at low monomer conversion (2–10%) when most of the monomer is in relatively large (1–10 μm diameter) droplets. Particle nucleation takes place when radicals formed in the aqueous phase from a (usually) water-soluble initiator grow via propagation and then enter surfactant micelles (*micellar nucleation)* or become large enough in the continuous phase to precipitate and form primary particles which may undergo limited flocculation until a stable particle population is obtained (*homogeneous nucleation*). Significant nucleation of particles from monomer droplets is discounted because of the small total surface area of the large droplets. Interval II involves polymerization within the monomer-swollen polymer particles with monomer supplied by diffusion from the droplets, across the aqueous phase, and into the polymer particles. Interval III begins when the droplets disappear and continues to the end of the polymerization. (This is an abstract model and may not be accurate in all or even most actual polymerizations; however, it provides a basis for understanding a very complex actual process. The product is a latex of submicron polymer particles in a continuous aqueous phase. The rate of polymerization, the particle size distribution in the final latex, and the molecular weight of the product polymer are all highly dependent on the ability of the monomer to diffuse into the polymer particles at a rate capable of sustaining polymerization. There are two major considerations in understanding the swelling of polymer particles by monomer: The equilibrium concentration of monomer in equilibrium with a continuous phase saturated with monomer, and the ability of the monomer to transport into the aqueous phase at a rate sufficient to support particle swelling equilibrium. These two key phenomena will be the subject of this paper. Before continuing, it is important to note that the monomer must transport out of the monomer droplets into the aqueous phase, across the aqueous phase, and into the polymer particles. The rate-limiting step in this process is taken to be the transport of monomer out of the monomer droplets. The aqueous phase is assumed well-stirred, and so the concentration of monomer in the aqueous phase is assumed to be equal everywhere. Monomer transport from the aqueous phase into the polymer particles occurs at some fixed rate, but given the relative diameters of the droplets (∼10 μm) and the particles (∼100–300 nm), and hence the even greater difference in surface areas for monomer transport, transport limitation into the particles is taken to be insignificant and transport out of the monomer droplets is assumed to be the rate-limiting step.

#### 1.1.2 Miniemulsion Polymerization

Miniemulsion polymerization involves the use of an effective surfactant/costabilizer system to produce exceedingly small (0.05–0.5 μm) monomer droplets. The droplet surface area in these systems is exceptionally large, and most of the surfactant is adsorbed at the droplet surfaces. Particle nucleation is primarily via radical (primary or oligomeric) entry into monomer droplets, since little surfactant is present in the form of micelles, or as free surfactant available to stabilize particles formed in the continuous phase. The reaction then proceeds by polymerization of the monomer in these small droplets; hence there is no true Interval II in an ideal miniemulsion. If an ideal miniemulsion can be defined as one in which all or nearly all the monomer droplets are initiated over a short time, there would be no issues of monomer equilibrium or transport. Since all polymer particles would start with identical size and monomer concentration, and would grow at the same rate, there would be no driving force for monomer transport between particles (and no monomer droplets). As with most idealizations, this one is not entirely accurate and the deviations from ideality will have significant ramifications in certain circumstances.

## 2 Monomer Equilibrium

### 2.1 Emulsion Polymerization

A conventional emulsion polymerization in Interval II has three separate phases: the continuous aqueous phase, the monomer droplets, and the polymer particles. These are commonly assumed to be in equilibrium. Thus, pure monomer in the monomer droplets of a homopolymerization will ensure that the aqueous phase is saturated with monomer. In a copolymerization of two or more monomers, the equilibrium monomer concentration of monomer *i i*n the aqueous phase will depend on the mole fraction of monomer *i* in the monomer droplets according to Raoult’s Law. Thus
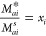
1where *M*_ai_^*^ is the aqueous monomer concentration (mol/L) in equilibrium with monomer droplets containing *x*_*i*_ mole fraction *i*, and *M*_*ai*_^*s*^ is the saturation concentration (mol/L) of monomer *i* in the aqueous phase (if exposed to pure *i*). If other, nonmonomeric species are present in the monomer droplets, their equilibrium aqueous phase concentration will follow the same relationship.

The more difficult task is predicting the concentration of monomer in the polymer particle in equilibrium with a given aqueous phase concentration. The rigorous solution is to use the Morton Equation^1,2^ This equilibrium relationship balances the Flory–Huggins free energy of the polymer swollen with monomer with the surface free energy of the small (highly curved) particle:
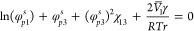
2

Here γ is the interfacial tension, φ_*p*1_^*s*^ is the volume fraction monomer in the particle, φ_*p*3_^*s*^ is the volume fraction polymer in the particle, χ_13_ is the Flory–Huggins interaction parameter for monomer (1) and polymer (3), *V̅*_1_ is the molar volume of monomer, *R* is the universal gas constant, *T* is the absolute temperature, and *r* is the radius of the particle. This equation captures the balance between swelling and interfacial area increase. The composition of the particle is a strong function of radius up to a particle radius of 20 nm, and a week function of particle radius thereafter, and is usually safe to assume a size-independent value for radii above 25 nm.^3^

If an additional component (component 2, e.g. second monomer, swelling agent, costabilizer or solvent) is added, Equation 2 has been extended by Higuchi and Ugelstad^4,5^ following Flory^6^ to
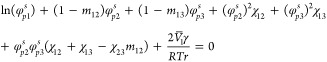
3

Additional variables here are φ_*p*2_^*s*^, the saturation volume fraction of component 2; *m*_12_ and *m*_13_, the ratios of molar volumes of component 1 to component 2, and component 1 to component 3 respectively; and χ_12_ χ_23_, the Flory–Huggins interaction parameters between components 1 and 2, and components 2 and 3, respectively. Approximating *m*_13_ as zero (since component 3 has already been assumed to be polymer), and setting φ_*p*2_^*s*^ equal to zero (no component 2) in Equation 3 gives Equation 2. The term containing γ accounts for the interfacial free energy. The terms containing *χ*_*ij*_(*i*, *j* = 1, 2 or 3) account for the enthalpy of mixing, and the terms containing only φ_*p*1_^*s*^ or *m*_*ij*_ and φ_*p*1_^*s*^ account for the entropy of mixing. Equation 2 is directly applicable for a binary copolymerization, with components 1 and 2 monomers. In this case, it must be written for monomer 1 and again for monomer 2. One can solve for the volume fractions of monomers 1 and 2, knowing that the remaining volume fraction is polymer. It will also be useful later in understanding miniemulsions.

Equation 3 allows for the determination of the composition of particles in equilibrium with an aqueous phase saturated with component 1 (monomer). If the aqueous phase is not saturated with monomer, Equation 3 becomes^7^
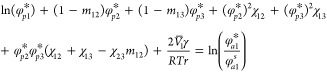
4

Here the superscripts *a* and *p* indicate the aqueous and particle phases, and φ_*a*1_^*^ and φ_*a*1_^*s*^ indicate, respectively, the actual and saturation volume fraction of component 1 in the aqueous phase. (Note that at saturation, the right-hand side of Equation 4 goes to zero.)

The use of Equations 2 – (4) requires the knowledge of the value of the interfacial tension (γ) between the particle and aqueous phases which depends on surfactant type and concentration, particle composition, and aqueous phase composition and may not be well-known. Values for the interaction parameters can be estimated,^8^ but the author has had little luck extracting reliable values by this method.

Equation 3 is semirigorous (as rigorous as Flory–Huggins Theory), but nonintuitive and difficult to use. For cases where the aqueous phase is not saturated, Gilbert^3^ recommends Equation 5 instead:
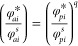
5

The value of *q* is taken to be 0.6 from experimental data. Here, of course, one must know φ_*p*i_^*s*^ from Equation 3 or experimental measurement. For copolymerization, one might apply Equation 5 independently for each monomer, assuming that the monomer concentrations in the aqueous phase are low enough that the monomers do not interact. (Recall that the aqueous phase will not be saturated with either monomer due to Raoult’s Law as shown in Equation 1. However, data for binary copolymerization^9,10^ indicate that Equation 5 may not hold with *q* = 0.6. In Table 1 the Mayo Lewis calculations (accounting for the reactivity ratios of the monomers) for the mole fraction of each monomer in the polymer (F_A_, F_B_) *assuming* the monomer ratio in the particle is equal to the monomer ratio in the droplet, agree well with the experimentally determined values, suggesting that *q* in Equation 5 ought to be 1.0 rather than 0.6. Samer^11^ and others have proposed the use of a partition coefficient, which amounts to using Equation 5 with *q* = 1.0.

**Table 1:** Copolymer Composition Estimated by the Mayo Lewis Equation Assuming the Monomer Ratio in the Particle is Equal to That in the monomer droplet Versus Actual Comonomer Composition

Monomers (A/B)	F_A_ Mayo Lewis Equation	F_B_ Mayo Lewis Equation	F_A_ Actual	F_B_ Actual
Acrylonitrile/Butadiene	0.38	0.62	0.35	0.65
Butyl Acrylate/Styrene	0.41	0.59	0.40	0.60
Vinyl Acetate/Methyl Acrylate	0.10	0.90	0.11	0.89
Vinyl Acetate/Butyl Acrylate	0.12	0.88	0.13	0.87

Maxwell and co-workers^12−15^ have developed an extension to the Morton Equation. One significant result of their work for binary copolymerization is give as
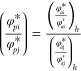
6where the subscript *h* indicates values for a homopolymerization. Asua and co-workers^10^ have compared the Morton, Maxwell and constant partition coefficient models for a wide range of experimental data. They found that different (of the three) models fit best when applied to batch and semibatch copolymerizations and copolymerizations where the commoners varied widely in water solubility.

Finally, in the absence of any better approach, Schork^16^ has recommended using 0.67 ± 0.04 for the saturated volume fraction monomer in a polymer particle. Since, as seen in Equation 3, swelling is driven primarily by entropic rather than enthalpic forces, it is not surprising that the saturation volume fraction monomer should be constant over a range of monomers and their polymers.

### 2.2 Miniemulsion Polymerization

#### 2.2.1 Role of Costabilizer

As noted above, miniemulsion polymerization depends on reducing the monomer droplet size to a point where the droplets become the primary locus of particle nucleation because of the large surface area of the small droplets and the lack of micelles, due to the surfactant adsorption onto the droplets. However, very small droplets are not stable due to Ostwald ripening.^1^ Ostwald ripening occurs when a small droplet is in the presence of a larger one. Monomer flows from the smaller droplet, across the aqueous phase, and into the larger droplet to reduce the total free energy of the system by reducing the total interfacial area. The effect can be quantified by writing Equation 2 for the large droplet and for the small droplet and setting them equal (equilibrating the system). The increase in surface area when the large droplet absorbs the diffusing monomer is less than the decrease in surface area when the smaller droplet loses the diffusing monomer due to simple volume-surface area considerations. This results in growth of the larger droplets and shrinkage of the smaller ones, and a reduction in the interfacial free energy. Ostwald ripening occurs very rapidly and will result in the eventual phase separation of the monomer from the aqueous phase. Small droplet stability against Ostwald ripening can be achieved by introducing a *costabilizer* (also referred to in the older literature as a cosurfactant or hydrophobe, although costabilizer is the accepted term based on the current understanding of its function). An ideal costabilizer should have very high solubility in the droplets and almost zero solubility in the aqueous phase (allowing no transport of the costabilizer out of the smaller droplet). Costabilizers that have been used successfully include cetyl alcohol, (CA) hexadecane (HD), highly water-insoluble commoners and initiators, alkyds, oligomers, and polymers. Thus, when a small droplet equilibrates with a larger one, the free energy reduction due to the overall reduction in interfacial area (and hence interfacial free energy) is offset by the free energy gain of the concentration the costabilizer in the smaller droplet. Ostwald ripening is eliminated, or at least greatly curtailed. Since the miniemulsion droplet contains a costabilizer, Equation 3 defines the droplet stability where component 1 is monomer, component 2 is the costabilizer, and component 3 is polymer (not yet present in monomer droplets). Equation 3 then becomes
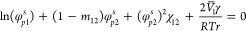
7

Here all terms containing φ_*p*3_^*s*^ have been eliminated since no polymer is present (except, perhaps, that being used as a costabilizer as discussed below). Equation 7 shows one more desirable attribute of a costabilizer. The symbol *m*_12_ represents the ratio of molar volumes of component 1 to component 2. For greatest reduction of Ostwald ripening, *m*_12_ should be large, meaning the molecular volume (and molecular weight) of the costabilizer should be low.^4^ Thus, CA, HD, and highly water-insoluble comonomers and initiators should be good costabilizers, while polymer, with a very high molecular weight, should be a poor costabilizer.

The first miniemulsions were reported by Ugelstad, El-Aasser and Vanderhoff in 1973.^17^ CA was used as the costabilizer, and the emulsifier was described as “an anionic emulsifier-fatty alcohol combination”. The fatty alcohol was CA, and very stable STY miniemulsions were made. Presumably, CA was used since it has some level of surface activity due to the hydroxyl group. In fact, early on, costabilizers were referred to as *cosurfactants*, suggesting some function as a surface-active agent. With cetyl alcohol, there is the complication that the polarity of the molecule may cause it to reside at the surface of the droplet, imparting additional colloidal stability. Here, the surfactant and costabilizer form an ordered structure at the monomer-water interface which acts as a barrier to coalescence and mass transfer. Results were very good, with very effective droplet stabilization and nucleation. This is supported by the fact that is necessary to pre-emulsify the surfactant solution with the CA before adding the monomer to form the ordered surfactant-CA structure. Ugelstad et al.^18^ continued the work with STY miniemulsions with CA as the costabilizer. Since these two papers, CA has been considered one of the two most effective stabilizers. In 1978 Ugelstad^4^ described the phenomenon of droplet stabilization as a retardation od Oswald ripening and suggested the use of HD or CA as the costabilizer. It should be noted that CA and HD vary only by one hydroxyl group, giving CA some surface activity and HD none. He also discussed the importance of low molecular weight of the costabilizer as discussed above. Hansen and Ugelstad^19^ showed that HD, in spite of its lack of surface activity, functioned very well as a costabilizer. Since then, HD has been the other most effective costabilizer; the choice of CA or HD is of little consequence.

Reimers and Schork^20^ used highly water-insoluble comonomers as costabilizers. Vinyl hexanoate, p-methyl STY, vinyl 2-ethyl hexanoate, vinyl decanoate, and vinyl stearate were copolymerized with MMA at ten weight percent on the total monomer All formed stable miniemulsions. All resulted in polymerization via predominant droplet nucleation. Chern and co-workers^21−24^ have used lauryl methacrylate and stearyl methacrylate as at levels of 2–3 wt % on the total monomer as both costabilizers and comonomers in the miniemulsion polymerization of STY. These high methacrylates have low water solubilities and high solubilities in STY monomer. The advantage of monomeric costabilizers is, of course, that, after polymerization, no low molecular weight costabilizer remains in the latex.

Mouran et al.^25^ polymerized miniemulsions of methyl methacrylate (MMA) with sodium lauryl sulfate as the surfactant and dodecyl mercaptan (DDM) as the costabilizer. The emulsions were of a droplet size range common to miniemulsions and exhibited long-term stability. Wang et al.^26^ successfully made miniemulsions of STY with DDM as the costabilizer. Since DDM is a low (relatively) molecular weight compound with very low water solubility and high monomer solubility, it is not surprising that it would be a good costabilizer.

Reimers and Schork^27^ have successfully used lauryl peroxide as both initiator and costabilizer in the miniemulsion polymerization of MMA. Schork and co-workers^28,29^ have used alkyds as costabilizers to form hybrid miniemulsions. Alkyds are the primary component of oil paints and most commonly consist of unsaturated natural oils extended with phthalic anhydride. Reactions catalyzed by oxygen from the air give a hard, cross-linked coating when the alkyd is applied as a film. Alkyds were added to miniemulsions of acrylic monomers at very high levels (up to 50 wt % of total solids), so, not surprisingly, the alkyds performed well as costabilizers in spite of their rather high molecular weight (500+ g/g-mol). It should be noted that the costabilizer function of the alkyd was secondary to the fact that it could be grafted on to acrylic polymer, during polymerization of the acrylic polymer, and then cross-link on exposure to air, providing a water-based Cross-linkable coating. Hence the high levels of the alkyd. Unfortunately, the miniemulsion particles tended to be heterogeneous, with the alkyd in the center and the acrylic on the surface and retarding cross-linking. This effect was somewhat alleviated by using unsaturated natural oils, or even unsaturated fatty acids in place of the alkyds.^30^ For the present purposes, it is only necessary to note that the oils and even fatty acids functioned well as costabilizers at the high levels necessary to provide sufficient cross-linking of the resultant polymer. In similar ways, unsaturated polyester^31^ and polyurethane^32^ provided adequate costabilizer function in spite of the extremely high molecular weight of the costabilizer, in part because they were used at levels of up to 50 wt % of total solids in order to provide a hybrid (grafted) polymer capable of cross-linking.

Schork and co-workers^33−35^ and have used polymer as a costabilizer. Polymer will not function as a highly effective costabilizer because, with its very high molecular weight, the value of *m*_12_ in Equation 3 would approach zero, severely degrading the effectiveness of the costabilizer. Ugelstad argued this point in 1978.^4^ However, if the polymeric costabilizer used is the same as the polymer to be produced in the miniemulsion, Equation 2 can be used. The term containing *m*_12_ does not appear in Equation 2. In addition, Ugelstad’s arguments were based on equilibrating costabilizer with pure, bulk monomer. This is not the case in a miniemulsion. If all of the monomer droplets are of the same radius and composition, there is no Ostwald ripening. If there is a variation in droplet size, and there will be, a small droplet should be equilibrated with a larger one (not bulk, pure monomer) to assess droplet monomer transport. In fact, if one writes Equation 2 for an MMA droplet containing 4 wt % poly(methyl methacrylate) (PMMA) and a radius of 100 nm and sets it equal to Equation 2 for a droplet of the same composition but with a radius of 150 nm, the equilibration results in the shrinkage of the smaller droplet to 80 nm radius, and the growth of the larger droplet to 159 nm radius. This is hardly Ostwald ripening and collapse of the miniemulsion. In a more severe test, one can equilibrate the 100 nm radius droplet with a 10 μm radius droplet typical of emulsion polymerization without the sonication or homogenization used in miniemulsion preparation. In this case, the smaller droplet shrinks to 46 nm, while the larger droplet grows to 10.07 μm. Again, this is limited monomer transport and broadening of the droplet size distribution, but not reversion to large emulsion (not miniemulsion) droplets. Thus, while polymer may not be the most effective costabilizer, it is capable of stopping Ostwald ripening and producing a thermodynamically stable miniemulsion. Two points can be made: First, if polymer is used as a costabilizer, the initial droplet distribution needs to be very. Second, as El-Aasser and co-workers have pointed out,^36,37^ polymer can preserve the number of miniemulsion particles, but not necessarily their size.

Miller et al.^38−40^ and Blythe et al.^36,37,41^ report that the addition of a small amount of polystyrene (PS) to the styrene phase of a miniemulsion polymerization of styrene caused an increase in both rate of polymerization and number of final polymer particles. This is not just a polymeric costabilizer effect, since these emulsions were also stabilized with what are known to be effective levels of HD. With the addition of 1 wt % styrene, the number of final particles was nearly the same as the original number of droplets, indicating 100% droplet nucleation. This was not the case for equivalent polymerizations without the PS. It was suggested that the enhancement can be primarily attributed to preservation of the droplet number by the presence of polymer in each of the miniemulsion droplets formed during homogenization. The authors rightly point out that the polymer is a poor costabilizer, due to its high molecular weight, but that it does act to preserve the droplet due to the thermodynamic balance between monomer and polymer. Thus, they conclude, as noted above, that polymer is unable to preserve the size of the droplets produced during ripening, but instead only the number produced during homogenization. This effect appears to be more pronounced in systems with CA (perhaps due to its polar nature, and so, its probable interfacial activity).

It should be noted that droplet stability does not need to be maintained for weeks or months, but only for the duration of the polymerization (hours). Thus true equilibrium stability is not necessary.

If droplet nucleation is near-complete in a miniemulsion, no monomer transport is necessary. If, however, droplet nucleation is substantially less than complete, then transport of monomer from the remaining monomer droplets to the growing polymer particles (as in emulsion polymerization) can be a significant event. Monomer transport is discussed below in Section 3.2.

#### 2.2.2 Reversible-Deactivation Radical Polymerization

Reversible-deactivation radical polymerization (RDRP), also known as controlled radical polymerization, is an alternative to ionic living polymerization for producing very narrow molecular weight distribution (MWD), and desired copolymer composition distribution CCD) and copolymer sequence distribution (CSD). This is accomplished by using a control agent to inactivate (without terminating) growing radical chains so that only a few chains are actively polymerizing at any given time. Since very few chains are live at any time, bimolecular termination is suppressed, and the lifetime of a radical chain goes from 1–10 s in free radical polymerization to hours or even months in CRP. This allows all chains to grow at the same rate, giving a very narrow MWD. Since the chains are live for long periods, it is also possible to add a second (or third) monomer after the existing monomer has been polymerized, allowing for the production of block copolymers (or other CCDs and CSDs other than those dictated by the Mayo Lewis Equation. Details of the chemistry and engineering of RDRP are available.^42,43^ For the current purposes a summary will do. There are three main chemistries for RDRP: *nitroxide-mediated controlled radical polymerization* (NMRP), *atom transfer radical polymerization* (ATRP) and *reversible addition–fragmentation transfer* (RAFT). In NMRP and ATRP the control agent (*T*) functions to deactivate the radical as
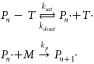
8

Here *P*_*n*_• is an active polymeric radical containing *n* monomer units. Since the first react ion above occurs very fast, each radical will exist in the active state for approximately the same cumulative time, and so will contain approximately the same number of monomer units *n*. RAFT operates by a different activations/deactivation chemistry:
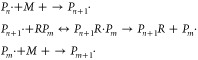
9

Here *R* is the control agent. For present purposes, it is only important to note that in all three chemistries, early in the polymerization a very large number of short oligomers will be formed. These oligomers have the properties of an excellent costabilizer: highly soluble in the droplets/particles, highly insoluble in the aqueous phase, and of low molecular weight (giving a high value of *m*_12_).

RDRP in miniemulsions[^44−49^ should provide colloidal stability since the control agents do not have to diffuse into the polymer particles as in conventional emulsion polymerization, and the control agents might also act as costabilizers. However, early CRP miniemulsions exhibited poor colloidal stability, large particle size and broad particle size distributions, as well as irreproducible particle size.^50−52^ As noted above, in a RDRP system, formation kinetics of a polymer chain is totally different from the free radical polymerization; in the very beginning of polymerization, few polymer chains, of high molecular weight are formed. Instead, a large number of oligomers are formed. Ugelstad et al. [40] showed that oligomers are very efficient swelling agents, and hence the existence of oligomers may dramatically modify the state of the miniemulsion.

A theoretical model has been developed by Luo et al.^53^ to simulate the swelling of oligomers formed in living miniemulsion polymerization and explain the resultant colloidal instability. In the case of the RDRP miniemulsion polymerization, during the early stages of polymerization a large amount of monomer would transfer from the un-nucleated droplets to the oligomeric polymer-containing particles, which would cause the monomer droplets to shrink and particles to swell. First, this would broaden the particle size distribution, or even destroy the miniemulsion. In the worst case, this *superswelling* would lead to a very large size difference between the droplets and particles. The particles could be swollen to around 1 μm, a critical size where the system become shear sensitive and buoyant forces dominate. Because in a miniemulsion polymerization reactor shear is low, the particles would rapidly approach a breaking–coagulating dynamic balance particle size, as in suspension polymerization. In this case, particle size could be more than 10 μm, or even form a bulk phase, depending on the shear fielded. Alternatively, it is possible to destroy the miniemulsion by the so-called heterocogualation (small particles/droplets coagulating onto large ones) when the size difference becomes large. Increasing the costabilizer level or using a polymeric surfactant are shown to be efficient ways to avoid superswelling.^52^ Both slow the transport of monomer from the droplets to the particles.

## 3 Monomer Transport

Section 2 above describes phase equilibrium and the approach to phase equilibrium in emulsion and miniemulsion polymerization. While the assumption of equilibrium simplifies the analysis of a multiphase process, it may not always be correct. In this section we will look at the possibility that the assumption of phase equilibrium is not valid. This is especially important since the development of miniemulsion polymerization, since monomer transport, if it is significant for a given monomer, is much *less* significant in miniemulsion polymerization than in conventional emulsion polymerization.

### 3.1 Emulsion Polymerization

#### 3.1.1 Batch Homopolymerization

The question to be investigated here is during Interval II of a conventional emulsion polymerization, is the rate of polymerization limited by monomer transport out of the monomer droplets, across the aqueous phase and into the polymerizing particles, or by the rate of polymerization within the particles. In 1953, Flory^6^ argued that emulsion polymerization of styrene was reaction limited, and since then this has been generally considered to be true for monomers that are at least as soluble in the aqueous phase as styrene. (A listing of monomer solubilities in water is given by Lovell and Schork.^1^) In the intervening years, various authors have wondered about possible mass transfer limitation with monomers much less water-soluble than styrene.

Reimers^20^ compared the copolymer composition versus overall monomer conversion profiles for various highly water-insoluble monomers for conventional emulsion and miniemulsion (likely to be less transport limited) polymerization. He speculated that the difference in copolymer composition profile between the two polymerization techniques was due to more severe transport limitations with conventional emulsion polymerization. With this reasoning, he found significant evidence of transport limitation for vinyl hexanoate, vinyl 2-ethyl hexanoate, vinyl decanoate, and vinyl stearate (VS). Back and Schork^54,55^ studied monomer-transport effects in the homopolymerization of two highly water-soluble monomers, isooctyl acrylate (IOA) and isobornyl acrylate (IBoA) by emulsion and miniemulsion polymerization and were not able to determine conclusively that emulsion polymerization was monomer-transport limited. Balic^56^ found reduced rates of polymerization and long inhibition periods for the emulsion polymerization of VEOVA-10. He attributed this to trace inhibitors but was unable to find them analytically. Schork and Back^57^ showed that for highly water-insoluble monomers (such as VEOVA-10) the low water solubility results in long residence times of the oligomers in the aqueous phase before they reach sufficient length to enter polymer particles. This results in extreme sensitivity to trace amounts of water-soluble inhibitors.

In order to investigate monomer transport during emulsion polymerization during interval II, Schork^58^ has drawn on the definition of the Damkohler Number. In reaction engineering the Damkohler Number is the ratio of the rate of reaction to the rate of transport of reactants to the reaction site. If both the reaction and transport rates are taken as their maxima, the Damkohler Number serves as a measure of reaction version transport limitation: if this Damkohler Number is greater than 0.1, the system is judged to be transport limited. For emulsion polymerization this Damkohler Number can be written as the rate of polymerization in the polymer particles assuming the particles are saturated with monomer, divided by the rate of monomer transport out of the monomer droplets, assuming the aqueous phase is totally devoid of monomer. The choice of the transport term requires some explanation. For an emulsion polymerization in Interval II, there are four possible rate-limiting monomer transport events. The first is the transport of monomer out of the monomer droplets and into the continuous aqueous phase. The second is transport across the aqueous phase. The third is transport from the aqueous phase into the polymerizing polymer particles. Finally, one could think of diffusion of monomer from the surface of the polymer particle to the center. Transport across the aqueous phase is discounted, since emulsion polymerizations are adequately stirred, and no monomer gradients are expected across the aqueous phase. Diffusion within the polymer particle is usually discounted on the argument that the particles are very small, and most polymerization takes place near the surface, rather than in the core of the particle. This leaves the two interphase transport events: diffusion out of the monomer droplets, and into the polymer particles. It is generally assumed that transport out of the monomer droplets is the rate-limiting transport event, since the total surface area of the particles is approximately 1000 times the total surface area of the monomer droplets. It will be further assumed that the polymer particles are in equilibrium with the monomer concentration in the aqueous phase, assuming equilibrium swelling of the particle with monomer. One should correctly use the Equation 2, balancing the free energy of swelling the polymer in the particle with the free energy associated with the increased particle-aqueous interfacial area. However, for the current purposes, the simpler form Equation 5 with q = 0.6 will be used. Equation 2 can be rewritten as
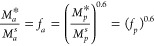
10where *M*_*a*_^*s*^ is the saturation value of the monomer concentration in the aqueous phase, *M*_a_^*^ is the actual concentration of the monomer in the aqueous phase, *M*_*p*_^*s*^ is the saturation value of the concentration of monomer in the particle in equilibrium with *M*_*a*_^*s*^, and *M*_p_^*^ is the actual concentration of monomer in the particle, in equilibrium with *M*_a_^*^. All concentrations are in mol/L. The symbols *f*_*a*_ and *f*_*p*_ are defined as the fractional monomer saturations of the aqueous phase and polymer particles, respectively. The Damkohler Number can then be defined as
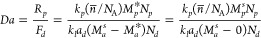
11

Here *F*_*d*_ is the rate of monomer transport out of the monomer droplets (mol/sec-L), *R*_*p*_ is the rate of polymerization (mol/sec-L), *k*_*p*_ is the rate constant for propagation, *n̅* is the average number of free radicals per particle, *N*_*p*_ is the number of particles per liter of emulsion, *N*_*A*_ is Avogadro’s Number, *k*_*l*_ is the mass transfer coefficient for monomer at the surface of the monomer droplet, *a*_*d*_ is the surface area of a droplet, and *N*_*d*_ is the number of monomer droplets per liter of emulsion. Values of *k*_*p*_ are available from the literature,^59^ or can be estimated from the class of monomer and its structure and molecular weight^58^ A value of 0.5 is assumed for *n̅* for all monomers for consistency. Values of *M*_*p*_^*s*^ are available in the literature,^3^ or, as Schork^16^ has pointed out, a value of 0.67 can be assumes for φ_*p*_^*s*^ for most monomers. The value of *k*_*l*_ is easily calculated, since, for droplets below 10 μm, the effects of Reynolds and Schmidt Number become insignificant, and *k*_*l*_ can be estimated as
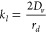
12where *D*_*v*_ is the diffusivity of monomer through the boundary layer in the aqueous phase around the droplet and *r*_*d*_ is the droplet radius and is assumed to be 5 μm.^33^ The value of *D*_*v*_ can be found from the Wilke Correlation.^60,61^ Again, monomer transport limitation is suspected if the value of Da is greater than 0.1. (Da equal to 0.1 corresponds to an aqueous monomer concentration that is 90 mol % of its saturation value. This gives a significant monomer transport limitation as well as accounting for the approximations made in the model above.)

Schork^58^ has calculated the Damkohler Number during Interval II of the emulsion polymerization of a wide range of monomers. A few common monomers, and those suspected of monomer transport limitation are listed in Table 2. (Monomers suspected of monomer transport limitation are shown in gray.). It should be noted that very few monomers are monomer transport limited. However, as Schork points out, as new functional monomers, macromonomers and biobased monomers are developed, it will be important to understand their transport limitations.

**Table 2:**
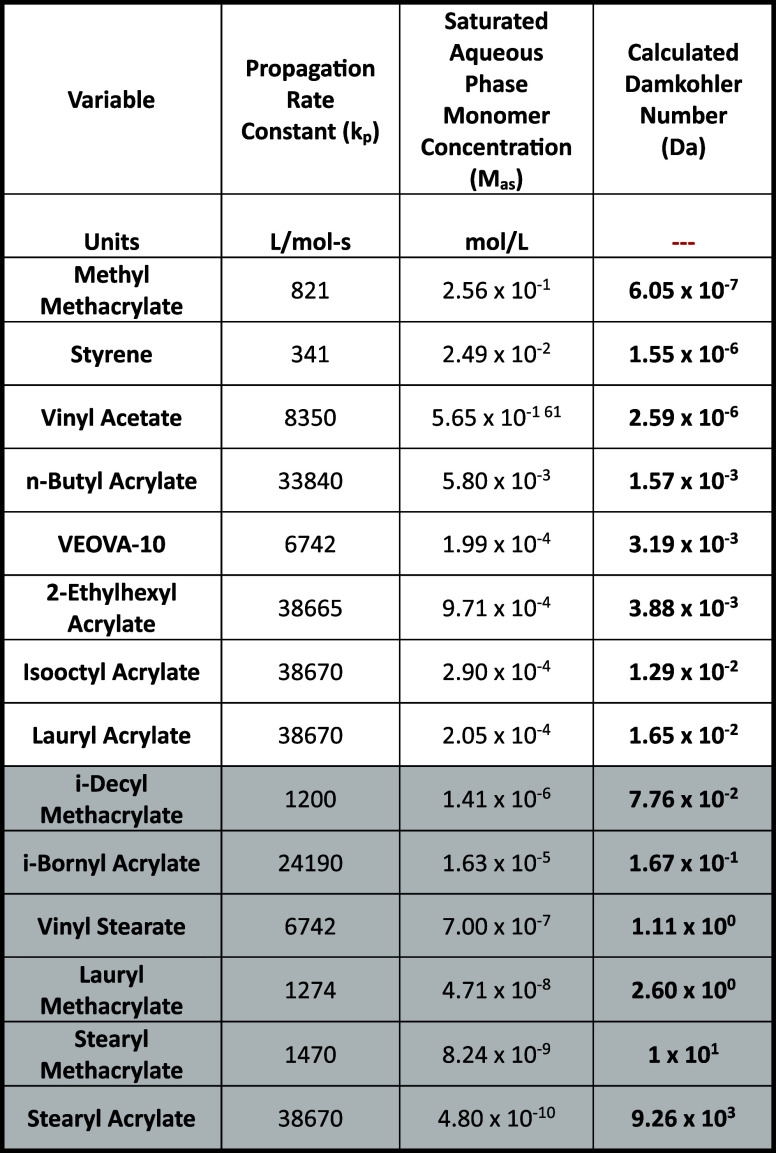
Damkohler Number for Selected Monomers^a^

a Shading indicates monomers with monomer transport limitation. From reference (58).

While calculating the Damkohler Number as described above takes some effort, Schork has pointed out that the value of the Damkohler Number is dominated by just two factors: the propagation rate constant and the monomer solubility in the aqueous phase. Based on this observation he suggest a short cut estimate for the Damkohler Number as
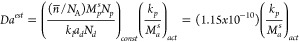
13which, for a 95% confidence interval for values of *Da*^*est*^ near 0.1 predicts that the actual *Da* to be between *0.84 Da*^*est*^ and *1.19 Da*^*est*^.

#### 3.1.2 Effect of Monomer Conversion on Monomer Transport Limitation

It should be noted that all Damkohler Numbers above were calculated at a fixed set of conditions. Specifically the monomer conversion for all monomers was assumed to be 30%. Referring to Equations 11 and (12) above, the mass transfer coefficient is proportional to the monomer droplet radius to the minus one power, while the area of a droplet is proportional to the monomer droplet radius squared. Thus, the Damkohler Number is proportional to the monomer droplet radius to the minus one power. This means that, as the monomer conversion increases and the monomer droplet radius decreases, the Damkohler Number will rise. In other words, near the point of the disappearance of the droplets, monomers that are not mass transfer limited may become so. This is shown in Table 3 below. Four monomers have been chosen, spanning the range of Damkohler Numbers in Table 2. Damkohler Numbers have been calculated as the monomer conversion increases toward the end of Interval II (denoted by the lack of data). It may be seen that STY remains monomer-transport unlimited, until the end of Interval II at about 40% conversion. The monomer 2-ethylhexyl acrylate (2-EHA) remains monomer-transport unlimited to the end of Interval II at approximately 37.4% conversion. IOA remains monomer-transport unlimited (but just barely) to the end of Interval II at 37.2% conversion. Decyl methacrylate (DMA) becomes monomer-transport limited somewhere between 30 and 35% monomer conversion.

**Table 3:** Damkohler Number as a Function of Monomer Conversion for Selected Monomers

Monomer Conv.	Da STY	Da 2-EHA	Da IOA	Da DMA
0.300	1.55 × 10^–06^	3.88 × 10^–03^	1.29 × 10^–02^	7.76 × 10^–2^
0.350	1.97 × 10^–06^	5.62 × 10^–03^	1.91 × 10^–02^	1.15 × 10^–1^
0.370	2.37 × 10^–06^	9.97 × 10^–03^	3.92 × 10^–02^	
0.372	2.43 × 10^–06^	1.22 × 10^–02^	6.45 × 10^–02^	
0.374	2.50 × 10^–06^	2.29 × 10^–02^		
0.376	2.58 × 10^–06^			
0.380	2.76 × 10^–06^			
0.390	3.70 × 10^–06^			
0.400				

#### 3.1.3 Batch Copolymerization

Most industrial polymers are copolymers, so most industrial emulsion polymerizations are copolymerizations. The previous section discussed monomer transport during homopolymerization, but a more relevant investigation might be, how does monomer transport limitation of one of two comonomers affect the copolymer composition of the resulting polymer? In a second paper on Damkohler analysis, Schork^62^ develops a method for calculating the Damkohler Number for a secondary monomer present at 10 mol % with a primary monomer. It is also assumed (based on common practice) that the secondary monomer is less soluble in the aqueous phase, and hence, possibly monomer-transport limited. It is assumed that equilibrium between the monomer droplets and monomer *B* in the aqueous phase (the secondary monomer) follows Raoult’s Law:
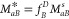
14where *M*_aB_^*^ is the concentration of monomer *B* in the aqueous phase at equilibrium, *f*_*B*_^*D*^ is the mole fraction of monomer *B* in the droplets, and *M*_*aB*_^*s*^ is the saturation concentration of monomer *B* in the aqueous phase (the concentration that would be in equilibrium with droplets of pure monomer *B*). The derivation makes the quasi-steady state approximation and follows the Mayo Lewis derivation for polymer composition. The result for the Damkohler Number for the secondary (less water-soluble) monomer *B* is
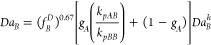
15where
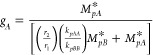
16and *r*_1_ and *r*_2_ are the reactivity ratios, *k*_*pAA*_ and *k*_*pBB*_ are the homopolymerization propagation rate constants, and *M*_pA_^*^ and *M*_pB_^*^ are the concentrations of monomers *A* and *B* in the particle in equilibrium with *M*_aA_^*^ and *M*_aB_^*^, obtainable by applying Equation 10 for each monomer. *Da*_*B*_^*h*^ is the Damkohler Number for the homopolymerization of monomer *B* as described in the previous section. Thus, with the knowledge of the homopolymerization Damkohler Number and all four propagation rate constants, it is possible to find the Damkohler Number for monomer *B* during copolymerization with monomer *A*. It should be noted that the Damkohler Number for monomer *B* is defined as the consumption of monomer *B* by polymerization divided by its transport; monomer *A* enters the calculation only through the relative reactivities of *A* and *B* radicals in adding *B* monomer. Since the copolymerizations Damkohler Numbers are based on the homopolymerization Damkohler Numbers, it is possible to write a shortcut (estimated) copolymerization Damkohler Number as
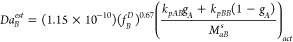
17

Damkohler Numbers for binary copolymerization pairs that indicate possible monomer transport limitation for the secondary monomer are listed in Table 4. In two cases (MA/DMA and DA/DMA) the level of secondary monomer limitation is greatly increased during copolymerization relative to homopolymerization. In all other cases, the level of monomer transport limitation is reduced in copolymerization relative to homopolymerization. In no cases studied does a monomer that is not transport limited in homopolymerization become limited in copolymerization.

**Table 4:**
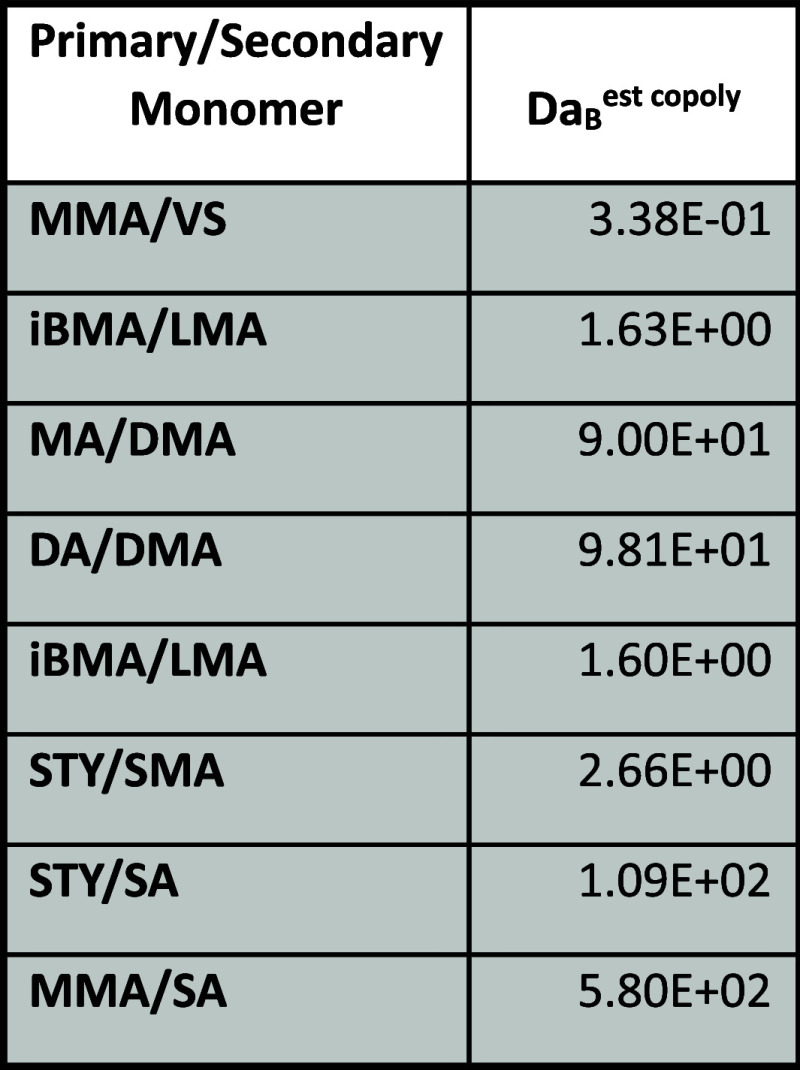
Damkohler Numbers for Binary Copolymerization Monomer Pairs Exhibiting Monomer Transport Limitation for the Secondary Monomers^a^

a Shading indicates monomers with monomer transport limitation. From reference (62). DA = decyl acrylate; DMA = decyl methacrylate; iBMA = isobutyl methacrylate; LMA = lauryl methacrylate; MA = methyl acrylate; MMA = methyl methacrylate; SA = stearyl acrylate; STY = styrene.

#### 3.1.4 Terpolymerization

Schork has extended the copolymerization treatment above to terpolymerization in which one of the monomers is suspected of monomer transport limitation. Since the derivation is mostly an exercise in algebra, it is not include here; details are available in the original paper.^63^

#### 3.1.5 Semibatch Polymerization

During a semibatch starved-feed copolymerization,^63^ monomer is added at a rate slow enough to ensure that the instantaneous copolymer composition is equal to the comonomer feed composition. In this case, it is unclear how much of the monomer exists in monomer droplets and how much is absorbed into the growing polymer particles. Under these conditions it makes sense to define the Damkohler Number for the monomer of lower water solubility (monomer *B*) under starved-feed conditions of both monomers as
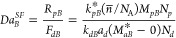
18

Here *F*_*dB*_ is the rate of monomer *B* transport out of the monomer droplets (mol/sec-L) *assuming all monomer is in the monomer droplets, rather than in the polymer particles*, *R*_*pB*_ is the rate of polymerization (mol/sec-L) of monomer *B*, *M*_*pB*_ is the monomer concentration in the particle, *assuming all monomer is in the particles, rather than in monomer droplets*, *k*_*dB*_ is the mass transfer coefficient for monomer *B* at the surface of the monomer droplet, and all other symbols are as previously defined. *k*_pB_* is a modified rate constant for propagation defined by
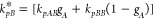
19Where *g*_*A*_ is the fraction of live chains ending in an *A* radical. The value of *g*_*A*_ may be found from Equation 16 above.

Assume that the primary monomer (*A*) is sufficiently water-soluble to ensure no monomer transport limitations, while the secondary monomer (*B*) is suspected of being monomer-transport limited. Assume that the that the instantaneous monomer conversion is held at 10% throughout the starved-feed portion of the copolymerization and that the secondary monomer is present in the overall monomer mix at 10 mol %. It can be shown^63^ that
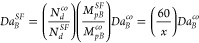
20where *Da*_*B*_^*co*^ is the batch copolymerization Damkohler Number previously discussed, x is the overall monomer conversion and all other symbols are as above, with the note that the superscript *co* refers to batch copolymerization, while the superscript *SF* refers to starved-feed copolymerization. The above indicates that under starved-feed conditions, *Da*_*B*_^*SF*^ will vary from 6 times *Da*_*B*_^*co*^ (10 wt % overall conversion) to 0.67 times *Da*_*B*_^*co*^ (90 wt % overall conversion). Thus, the condition of highest Damkohler Number (most likelihood of monomer *B* transport limitation) will be at the earliest stages of the semibatch period.

#### 3.1.6 Polymerization of Gaseous Monomers

Equation 11 was modified for gaseous monomers by Merlin and Schork.^64^ Since there are no droplets of the gaseous monomer, but only gas bubbles, the term (*k*_*l*_*a*_*b*_) is substituted, where (*k*_*l*_*a*_*b*_) is the product of the mass transfer coefficient at the selected operating conditions multiplied by the area of gas bubbles per cm^3^ of emulsion. The Damkohler Number for the homopolymerization of gaseous monomers results in the following:
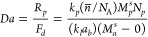
21

Ecoscia et al. carried out polymerizations of vinylidene fluoride (VDF) at a pressure of 88 bar (8,800 kPa), a temperature of 60 °C, and a stirring rate of 650 RMP.^65,66^ At these conditions, Ecoscia et al.^65^ reported a value of 0.47 min^–1^ for *k*_*l*_*a*_*b*_ for VDF emulsion homopolymerization. The mass transfer coefficients for all the other monomers were calculated by adjusting the VDF coefficient as follows:^64^
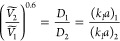
22

Here *Ṽ*_*i*_ is the specific volume of monomer *i, D*_*i*_ Is the diffusivity of monomer *i* in water, and (*k*_*l*_*a*_*b*_)_*i*_ is the product (*k*_*l*_*a*_*b*_) for monomer *i* under the operating conditions described above. *M*_*a*_^*s*^ was calculated using Henry’s Law constants from the literature. Since some of the gaseous monomers considered are liquids or supercritical at 8,800 kPa, pressures of 880 and 101.3 (1 atm) were also considered. All other values are identical to those for the liquid monomer Damkohler analysis.^58^

Selected Damkohler Numbers for gaseous monomers batch homopolymerized are listed below in Table 5. As with all the Damkohler Number calculations, this is meant to provide a set of identical conditions under which various monomers can be compared. As might be expected, the value of the Damkohler Number (and the propensity for monomer transport limitation) depends strongly on the pressure of the reaction.

**Table 5:** Selected Damkohler Numbers for Batch Homopolymerization of Gaseous Monomers From Reference (64)

Monomer	Damkohler Number	Damkohler Number
	880 kPa	101.3 kPa
Vinylidene Fluoride	0.134	1.16
Tetrafluoroethylene	4.12	35.8
Vinyl Chloride	0.509	4.42

Merlin and Schork^64^ have also investigated copolymerizations in which one of the comonomers is gaseous and suspected of monomer transport limitation, and the other is liquid. In this case Equations 15 and (16) can be used, with *Da* from Equation 21 substituted for *Da*_*B*_^*h*^. Selected values of the Damkohler Numbers for gaseous monomers (10 mol %) copolymerized with liquid monomer (90 mol %) are listed in Table 6. Again Da is strongly dependent on reactor pressure and values of *Da* explain why some polymerizations of gaseous monomers must be operated at extremely high pressures.

**Table 6:** Selected Damkohler Numbers for Batch Copolymerization of Gaseous Monomers with Liquid Monomers From Reference (64)

ComonomersGaseous/Liquid	Damkohler Number	Damkohler Number
	880 kPa	101.3 kPa
**Vinyl Acetate/Ethylene**	0.018	1.53
**Methyl Methacrylate/Vinyl Chloride**	0.0001	0.006
**Styrene/Vinyl Chloride**	0.027	2.33

### 3.2 Miniemulsion Polymerization

In an ideal miniemulsion, where all droplets are nucleated into particles, monomer transport would be of little or no importance. However, in actual miniemulsions, droplet nucleation is less than complete, and monomer transport from unnucleated monomer droplets to nucleated, growing polymer particles can be significant. Without knowing the extent of droplet nucleation, it is hard to quantify any monomer transport limitation, however it would qualitatively follow the description above for conventional emulsion polymerization.

### 3.3 Monomer Transport via Collision

Smeets^67^ observed mass transfer of the extremely water-insoluble chain transfer agent COPhBF during emulsion polymerization and attributed it to a collision mechanism. Tauer and co-workers^68,69^ have argued for significant collision-based monomer transport in emulsion polymerization. Anecdotal wisdom describes emulsion polymerization of extremely water-insoluble monomers in which the process reverts to a microsuspension polymerization, polymerizes in a bulk phase causing massive coagulation, or fails to polymerize at all.

El-Aasser and co-workers^70−72^ saw mass transfer limitations for monomer, costabilizer and oil soluble initiator in miniemulsion polymerization and postulated droplet-particle collisions as a mode for overcoming transport limitations. Jansen et al.^73^ studied the miniemulsion copolymerization of highly water-insoluble monomers lauryl methacrylate and 4-tertbutylstyrene with MMA, and postulated that what monomer transport is occurring (because not all droplets become particles as in the idealized concept of a miniemulsion) with the highly water-insoluble comonomers, occurs via droplet–droplet and droplet-particle collisions. They too postulated that collision is a significant mechanism of mass transfer for copolymerization, “Mass transfer that results in the formation of a copolymer in the miniemulsion polymerization of a mixture of lauryl methacrylate and 4-tertbutylstyrene, is almost non-existing when physical contact between individual droplets is prevented by a membrane.” Smeets et al.^74^ observed mass transfer of the extremely water-insoluble chain transfer agent COPhBF and attributed it to a collision mechanism. As noted above, Reimers^20^ found that miniemulsion copolymerizations followed the Mayo Lewis Equation for copolymer composition (indicating no monomer transport limitation) while the copolymer composition for conventional emulsion copolymerizations showed large deviations (indicating significant monomer transport limitation).

The monomer (and other) transport from one droplet/particle to another based on gradients is possible during miniemulsion polymerization, but limited compared with emulsion polymerization. Under these conditions of limited transport via the conventional interphase transport, it is likely that collision may become important for monomer and chain transfer agent transport as shown References.^73,75^ In summary, it would appear that collision transfer is happening to some extent in all systems, but only when conventional interphase transport becomes limited (by low water solubility or the presence of swelling agents) does it become significant. Alex van Herk has recently published a good perspective on transport via collision.^76^

## 4 Summary and Conclusions

In summary, one may see that monomer equilibrium among the monomer droplets, polymer particles and aqueous phase is best described by the Extended Morton Equation (Equation 4]. While it does give insights into the entropic and enthalpic effects in particle swelling, this equation requires parameters that are not easily obtained. However, it should be used whenever possible. Simpler models exist [Equations 5 and (6) and *ϕ*_*m*_ = 0.67] and may be used in the absence of a rigorous treatment.

Miniemulsion droplets must be stabilized against Ostwald ripening. The best costabilizers are HD, CA and highly water-insoluble comonomers. Preformed polymers at high levels will stabilize hybrid miniemulsion droplets. Polymer at low levels is a costabilizer, but a poor one unless the droplet distribution is very narrow. The effectiveness of a costabilizer can be estimated using the extended Morton Equation. Controlled radical polymerization in miniemulsions are especially vulnerable to colloidal instability due to superswelling brought on by the extremely large number of oligomers in the polymer particles in the early stages of polymerization. Superswelling can be combated by the use of additional costabilizer, rapid initiation, and polymeric surfactant.

The formalization of a Damkohler Number for emulsion polymerization can be used to identify monomers with potential monomer transport limitations. This approach can be applied to homopolymerization, copolymerization and terpolymerization in batch, starved-feed and gaseous-monomer polymerizations. Based on this analysis *most* monomers are *not* mass transfer limited, *but*, as new functional monomers, macromonomers and biobased monomers are developed, it will be important to understand their transport limitations.

One additional use of the Damkohler Number would be when selecting a monomer as a model compound. In cases where the actual monomer is extremely expensive, highly toxic, highly proprietary, it might be desirable to carry out preliminary studies with a model monomer exhibiting similar reactor properties. This model compound could be chosen as one with a similar Damkohler Number.
